# Reliability and validity of the perceived variety support in physical education scale among chinese school-attending adolescents

**DOI:** 10.3389/fpsyg.2026.1805418

**Published:** 2026-08-03

**Authors:** Huiying Fan, Shiyun Chen, Guoqiang Fan

**Affiliations:** 1 School of Physical Education, Shanghai Normal University, Shanghai, China; 2 Department of Applied Social Sciences, The Hong Kong Polytechnic University, Hong Kong, Hong Kong SAR, China; 3 Department of Sports, Shanghai University of Traditional Chinese Medicine, Shanghai, China

**Keywords:** Chinese adolescents, perceived variety support, physical education, reliability and validity, scale validation

## Abstract

In recent years, research on perceived variety support in physical education has received increasing attention. There is a clear need to translate the Perceived Variety Support in Physical Education Scale (PVSPES) into other languages. This study translated the PVSPES scale into Chinese and examined its reliability and validity through empirical analysis. A total of 1,182 high school students (mean age = 16.04 ± 0.9 years) from six schools in Shanghai, China, participated in the study. All participants completed the PVSPES questionnaire. Exploratory factor analysis showed that the data were well suited for factor analysis (KMO = 0.942; Bartlett’s test of sphericity, *p* < 0.001). The results supported a single latent factor model, with item factor loadings ranging from 0.68 to 0.86. The confirmatory factor analysis supported a single-factor solution (CFI = 0.97, TLI = 0.96, RMSEA = 0.08). The scale was also validated across different gender groups. It showed excellent internal consistency (Cronbach’s α = 0.93) and strong test–retest reliability (ICC = 0.94). These results provide initial evidence for the validity and reliability of the Chinese version of the PVSPES in the present sample of Chinese high school students.

## Introduction

Regular physical activity is important for the physical and mental health of children and adolescents. Recent evidence indicates that regular physical activity is associated with lower risks of cardiovascular disease, type 2 diabetes, several cancers, and premature mortality, while also contributing to better mental health and lower risk of depression(Garcia et al., 2023; World Health Organization, 2022; Pearce et al., 2022). China has a large school-aged population, but many Chinese adolescents do not meet recommended levels of physical activity (Liu et al., 2023). Research evidence indicates that many children and adolescents in China engage in insufficient levels of physical activity during the school years (Qin et al., 2026). This is concerning because physical inactivity insufficiency not only poses a threat to the health of school-age students in the present, but also as they enter adulthood, their PA tend to remain or decline, which can pose a continuing threat to their health in adulthood (Husøy et al., 2024; Miller et al., 2024).

In China, schools require all students to take part in physical education (PE) as a core element of the academic program from the first year of primary education through the second year of university, and it is also assessed as an examination subject in the majority of schools. These curricula are designed not only to encourage exercise participation but also to support students’ broader development. At the primary and secondary levels, the central goal of physical education is to foster healthy physical, psychological, and social growth among students (Eryong and Li, 2020). School-based PE allows teachers to explain the health benefits associated with regular physical activity. It also helps students acquire fundamental motor skills that support independent participation outside school. In addition, PE classes expose students to diverse forms of physical activity. These experiences encourage regular engagement in movement across the school years (Cale, 2023). As PE curriculum are typically mandatory in schools and taught by qualified instructors, they offer a distinctive and valuable opportunity for all young people to acquire skills and foster development.

Self-determination Theory (SDT) suggests that an individual’s behavior is influenced by the need for competence, autonomy, and relatedness, and researchers have validated the SDT’s explanations of an individual’s behavior (Teixeira et al., 2012). With further research, Sylvester et al., extends SDT in that the variety support can be used as a complement to the three aspects of SDT to influence individuals’ motivation and behavior (Sylvester et al., 2014). Scholars have described variety as the presence of multiple activities, options, or challenges within a specific setting. Recent research has extended this concept within exercise contexts. Variety support refers to the experience of engaging in a range or alternation of tasks, actions, and opportunities, whether novel or familiar. Variety can be conceptualized both as a characteristic of an activity or environment (i.e., variety support) and as a subjective experience (i.e., an individual’s felt experience). Variety in physical activity experiences may reduce monotony and promote more positive psychological responses, including enjoyment, interest, and positive affect (Tilga et al., 2025). Previous research has shown that participation in a wider range of physical activities is associated with greater physical activity enjoyment and participation among adolescents (Michael et al., 2016). Previous research has shown that offering a greater range of activities can positively influence individuals’ responses to physical activity. Increasing the type of physical activity prescribed and the variety of physical activity equipment available can increase physical activity as well as interest in physical activity. Research has demonstrated that expanding the range of exercise equipment can shape individuals’ activity experiences across different age groups. Access to sports equipment may facilitate adolescents’ participation in physical activity and indirectly support activity engagement through greater physical activity enjoyment (Burns et al., 2022). Participants exposed to high levels of variety support exhibited greater adherence to exercise compared with those assigned to the low variety support condition (Sylvester et al., 2015).

As an organization of physical activity in a specific form, the support of variety in PE curricula is considered to be one of the influencing factors in promoting students’ participation in physical activity in class and stimulating their interest in sport (Bantjes et al., 2015; Jago et al., 2015). Integrating variety into physical education may promote more positive emotional experiences during participation (Biddle et al., 2019). Using varied teaching methods may help reduce boredom and increase students’ interest and engagement in PE lessons. Recognition of the role of variety in physical education has spurred efforts to measure this concept more accurately. To this end, Abós and colleagues introduced a measure in 2021. This scale consists of five items and aims to assess students’ perceived variety in physical education (PTVPE) (Abós et al., 2021). Application of the scale to samples of adolescents showed acceptable levels of validity and reliability. Empirical research has also revealed that adolescents who perceive greater variety in physical education classes exhibit higher levels of intrinsic motivation. In a sample of 908 students, perceived variety explained a meaningful proportion of the variance in autonomous motivation during physical education classes. Despite these contributions, the prior PTVPE study did not address several key aspects of the physical education context that may enhance variety. Elements such as the use of different equipment, instructional approaches, student grouping strategies, and available facilities were not included in the analysis (Abós et al., 2021). Given that exposure to diverse physical activity settings (such as physical education classes, sports participation, leisure-time exercise, and gym-based activities) may act as a psychological driver of enhanced motivation and well-being in adolescents, Eather et al. (2022) introduced the Perceived Variety Support for Physical Education Scale (PVSPES). Designed for implementation in both observational and intervention-based studies, the scale comprises eight items that evaluate students’ perceived variety supportive features of PE classes, including variation in activities, teaching methods, and environmental elements (Eather et al., 2022). Eather’s study invited 20 experts to review the assessment, and the results validated the validity of the PVSPES scale for use with adolescents, and the scale’s measurement invariance and test-retest reliability were validated.

PE curricula is an important way to promote the overall development and healthy growth of Chinese children and adolescents. The China Physical Education Curriculum Standards emphasize a health-oriented teaching philosophy and aim to cultivate students’ core competencies through diverse, student-centered learning experiences (Ministry of Education of the People’s Republic of China, 2022). This orientation is highly consistent with the concept of variety-supportive physical education, which captures students’ perceptions of variation in lesson organization, activity content, grouping arrangements, use of space, and instructional approaches. However, before the PVSPES can be applied with confidence in Chinese physical education research and practice, its Chinese version requires systematic psychometric evaluation. Such validation is essential for supporting the wider use of the PVSPES among Chinese students. Therefore, the main research purposes of this study were adapted the PVSPES into Chinese and provided evidence for its validity and reliability.

## Materials and methods

### Participants

Participants in this study were 10th, 11th, and 12th grade students in Shanghai, China. During the study, we adopted the convenience sampling method to select 3 urban districts and 3 suburban districts (urban districts: Jing’an District, Yangpu District, Huangpu District; suburban districts: Pudong New District, Qingpu District, Minhang District), and then contacted one high school in each of these districts. One class of students from each high school was sampled from each grade. A total of 1,356 students from the six schools agreed to participate and returned questionnaires. During data screening, 174 questionnaires were excluded because of incomplete responses, obvious regular response patterns, or withdrawal from the study. The final valid sample included 1,182 students, corresponding to a valid response rate of 87.17%. The mean age was 16.04 ± 0.9 years, 46.9% (554 out of 1,182) were boys. According to the standards (Anthoine et al., 2014; Santos et al., 2015), samples of about 500 are regarded as very strong. To ensure independent exploration and validation of the factor structure, the total sample was randomly divided into two independent subsamples. Sample 1 (*n* = 608) was used to conduct the exploratory factor analysis (EFA), and Sample 2 (*n* = 574) was used to perform the confirmatory factor analysis (CFA) of the structure identified in the EFA. To assess the test–retest reliability of the scale, 349 students (182 boys and 167 girls) were randomly selected from a high school and completed the scale twice, with a 2-week interval between administrations.

### Procedures

The head of physical education in the selected schools was informed about the purpose and procedures of the study prior to the survey. Subsequently, physical education teachers and researchers jointly introduced the project to the students. Students who declined participation were asked to withdraw, while those who agreed were required to obtain written consent from their guardians. All students who took part in the project had obtained consent from their guardians. Throughout the entire testing process, all adolescents participating in this project have provided informed consent from both themselves and their guardians. Before initiating the survey, researchers will reassess students’ willingness to participate; those who decline may leave voluntarily. During the questionnaire completion process, researchers provided full assistance, and students could receive responses from researchers to any questions they had regarding the questionnaire. Students completed the questionnaire without providing identifying information. The study also received formal ethical clearance. The Ethical Review Board of Shanghai Normal University approved the research protocol under approval number 2023079.

### PVSPES

The PVSPES consists of eight questions that investigate the level of perceived support for equipment, space, opportunity, content, objectives, teaching style, peer skill support, and form of activity in physical education. Respondents chose to answer using four options ranging from ‘never’ to ‘always’ (Eather et al., 2022). Higher frequencies indicate higher levels of perceived support in the PE class.

### Translation of PVSPES

We obtained permission to use PVSPES from the original author. Forward and reverse translations were performed to confirm accuracy. The scale was first translated independently into Chinese by the first author of this study, and the reverse translation from Chinese to English was conducted by another researcher specializing in sport who was not involved in the initial translation. Three rounds of forward and reverse translations were conducted by the two researchers, and finally a college English major compared the original English text with the back-translated English version, and the three resolved inconsistencies through a consensus meeting. After three rounds of forward and backward translation and validation, the final version was validated by three middle and high school teachers. The Chinese version was finalized with no disputes or new suggestions, details of the PVSPES and its Chinese expressions are provided in Table 1.

**TABLE 1:** Original content of PVSPES and content of the Chinese version.

Measures	Original content of PVSPES	Content of the Chinese version
Item 1	In PE, my teacher provides a range of different sports equipment for me to use across the term (e.g., bats, balls, hoops, racquets, nets)	在整个学期的体育课中,我的体育老师提供了不同的运动器材供我在体育课上使用(例如,球类、呼啦圈、球拍)
Item 2	My PE teacher takes us to different locations within our school (to run different types of lessons) in PE class (e.g., gym, oval, hall, basketball court)	在不同类型的体育课中,我的体育老师会带我们到学校内的不同地点上体育课(例如,操场、篮球场、室内体育馆)
Item 3	In PE, I get to try a range of activities during one lesson.	在体育课上,我有机会在一节课中尝试进行一系列的活动
Item 4	In PE, activities vary from one lesson to the next	在体育课上,每节课的内容随着课程需要的变化而变化
Item 5	In PE, the goal of activities is varied (e.g., to improve skills or fitness, to work as a team, to score points, to improve my decision making)	在体育课上,活动目标是多样的(例如,提高运动技能或体能、培养团队合作能力、提高决策能力等
Item 6	In PE, I notice my teacher using different ways to teach me things if I don’t understand (e.g., teacher tells me or demonstrates how to perform a skill)	在体育课上,当遇到我不理解的内容时,体育老师会使用不同的方式教导我,(例如,老师会向我讲解或示范某一技术动作)
Item 7	In PE, I get to work with people with different skill levels (e.g., beginner, advanced)	在体育课上,我可以与不同技能水平的人一起活动（例如,初学者、熟练者)
Item 8	In PE, activities are delivered in lots of different ways (e.g., team games, pairs, circuit)	在体育课上,各种活动以许多不同的形式开展（例如,团队游戏、双人配合、循环练习)

### Motivation in PE

Students’ motivation in PE were measured using four subscales from the Chinese version of the Perceived Locus of Causality Scale in physical education (PLOC) (Goudas et al., 2011). The four subscales assessed intrinsic motivation, identified regulation, external regulation, and amotivation. Participants responded to each item on a 7-point Likert scale ranging from 1 (strongly disagree) to 7 (strongly agree). Previous research has supported the reliability and validity of the Chinese version of the PLOC among Chinese school students (Yang et al., 2019). In the present study, scores for each subscale were calculated by averaging the relevant items.

### Analyses

To facilitate scale development and validation, the total sample was randomly divided into two independent subsamples. Sample 1 was used for EFA to identify the underlying factor structure, whereas Sample 2 was used for CFA to validate the factor structure identified in the EFA.

Prior to conducting the EFA, descriptive statistics, including item means, standard deviations (SDs), and inter-item correlations, were examined in Sample 1. Sampling adequacy was evaluated using the Kaiser–Meyer–Olkin (KMO) measure and Bartlett’s test of sphericity. Adequate KMO values and a significant Bartlett’s test indicated that the data were suitable for factor analysis. KMO values ≥ 0.80 and a significant Bartlett’s χ^2^ (*p* < 0.001) indicated suitability for factor analysis.

EFA was conducted in Sample 1 using principal axis factoring with Promax rotation, as correlations among latent factors were expected. The number of retained factors was determined based on the screen plot, eigenvalues greater than 1, and theoretical interpretability (Costello and Osborne, 2005). Items were retained according to their factor loadings and conceptual relevance.

The factor structure identified in the EFA was subsequently evaluated in Sample 2 using CFA. Model fit was assessed using multiple goodness-of-fit indices, including the comparative fit index (CFI), Tucker–Lewis index (TLI), root mean square error of approximation (RMSEA), and standardized root mean square residual (SRMR). Acceptable model fit was indicated by CFI and TLI values of 0.90 or above, RMSEA values below 0.08, and SRMR values below 0.08 (Hu and Bentler, 1999). Standardized factor loadings were examined to evaluate the contribution of each item to its corresponding latent factor.

To evaluate measurement invariance across gender, multi-group CFA was conducted in Sample 2 to test configural, metric, and scalar invariance. Measurement invariance was supported when changes in model fit indices met the recommended criteria of ΔCFI ≤ 0.010 and ΔRMSEA ≤ 0.015 across nested models.

Convergent validity was evaluated using the average variance extracted (AVE) derived from the confirmatory factor analysis. An AVE value of 0.50 or higher was considered indicative of adequate convergent validity. Criterion-related validity was examined by assessing the association between PVSPES scores and students’ motivation in physical education, measured using the Chinese version of the Perceived Locus of Causality Scale (PLOC). Pearson correlation analysis was conducted to examine the relationship between the two measures.

Internal consistency reliability was evaluated using McDonald’s omega and Cronbach’s alpha coefficients. Higher values indicated greater consistency among items measuring the same latent construct. Test-retest reliability was assessed using intraclass correlation coefficients (ICCs) based on data collected from participants who completed the scale twice with a 2-week interval between administrations.

## Results

### Descriptive analyses

Table 2 presents the descriptive characteristics and bivariate correlations of the questionnaire items. On the four-point scale, item means ranged from 2.83 to 3.17. Correlation analysis showed that all items were positively correlated with each other. All correlations were statistically significant. Pearson correlation coefficients ranged from 0.50 to 0.73, indicating moderate to strong associations among the items.

**TABLE 2:** Means, standard deviations, correlation coefficient, skewness and kurtosis of each item (*N* = 1182).

Measures	Item 1	Item 2	Item 3	Item 4	Item 5	Item 6	Item 7	Item 8
Item 1	1	1	1	1	1	1	1	1
Item 2	0.67***							
Item 3	0.59***	0.63***						
Item 4	0.62***	0.63***	0.68***					
Item 5	0.60***	0.64***	0.68***	0.73***				
Item 6	0.60***	0.67***	0.62***	0.69***	0.73***			
Item 7	0.50***	0.54***	0.56***	0.53***	0.60***	0.61***		
Item 8	0.58***	0.63***	0.61***	0.64***	0.70***	0.69***	0.64***	
Mean	3.05	2.83	3.06	3.14	3.05	3.03	3.17	2.98
Standard deviation	0.99	1.04	0.93	0.94	0.96	0.97	0.92	0.98
Kurtosis	−0.98	−1.32	−1.03	−0.74	−0.94	−0.90	−0.41	−1.06
Skewness	−0.55	−0.19	−0.46	−0.66	−0.53	−0.53	−0.80	−0.42
Corrected item–total correlations (CITCs)	0.72	0.76	0.76	0.78	0.82	0.80	0.68	0.78

***Statistically significant at *p* < 0.001.

Table 2 also reports the skewness, kurtosis, and Corrected item–total correlations (CITCs) of item responses. All skewness and kurtosis values fell within the range of -1.5 to 1.5, suggesting that the item responses were approximately normally distributed and suitable for subsequent factor analyses. CITCs ranged from 0.68 to 0.82, indicating strong associations between individual items and the overall scale score.

### Exploratory factor analyses

The Kaiser–Meyer–Olkin (KMO) measure of sampling adequacy was 0.94, and Bartlett’s test of sphericity was significant, χ^2^(28) = 3022.81, *p* < 0.001, indicating that the data were suitable for factor analysis. Inspection of the scree plot and eigenvalues supported a one-factor solution. The extracted factor had an eigenvalue of 5.30 and accounted for 66.30% of the total variance. The factor loadings of the eight items ranged from 0.73 to 0.86, indicating that all items contributed substantially to the latent construct. Therefore, all eight items were retained for subsequent analyses.

### Confirmatory factor analysis

CFA was conducted on Sample 2 to evaluate the one-factor structure identified in the EFA. The hypothesized one-factor model demonstrated an acceptable fit to the data, χ^2^(df) = 101.55(20), *p* < 0.001, CFI = 0.97, TLI = 0.96*, RMSEA = 0.08[90% CI: 0.068, 0.101]*, and SRMR = 0.03. All standardized factor loadings were statistically significant (*p* < 0.001) and ranged from 0.68 to 0.85, indicating that each item contributed substantially to the latent construct. The standardized factor loadings are presented in Table 3.

**TABLE 3:** Standardized factor loadings and residual variances.

Item	Standardized factor loadings
Item 1	0.73
Item 2	0.77
Item 3	0.81
Item 4	0.85
Item 5	0.80
Item 6	0.83
Item 7	0.68
Item 8	0.82

### Validity analyses

Convergent validity was supported by an average variance extracted (AVE) value of 0.61, exceeding the recommended threshold of 0.50. Criterion-related validity was supported by a significant positive correlation between PVSPES scores and motivation in physical education as measured by the PLOC (*r* = 0.365, *p* < 0.001). This finding was consistent with theoretical expectations and previous research suggesting that greater perceived variety support is associated with more adaptive motivational outcomes in physical education.

### Subgroup analyses

Measurement invariance across gender was examined using multi-group confirmatory factor analysis. As shown in Table 4, the configural invariance model demonstrated an acceptable fit to the data, χ^2^(56) = 161.39, CFI = 0.966, TLI = 0.956, RMSEA = 0.081, and SRMR = 0.038.

**TABLE 4:** Measurement invariance results for gender.

Model	χ^2^	df	CFI	TLI	RMSEA	SRMR	ΔCFI	ΔRMSEA
Configural	161.39	56	0.966	0.956	0.081	0.038	–	–
Metric	169.63	63	0.965	0.96	0.077	0.048	0.001	0.004
Scalar	191.66	70	0.96	0.959	0.078	0.055	0.005	0.001

CFI, comparative fit index; TLI, Tucker–Lewis Index; RMSEA, root mean square error of approximation; SRMR, standardized root mean square residual. ΔCFI and ΔRMSEA are calculated relative to the preceding model. Measurement invariance is supported when ΔCFI ≤ 0.010 and ΔRMSEA ≤ 0.015.

Subsequently, metric invariance was tested by constraining factor loadings to be equal across gender groups. The metric invariance model also demonstrated acceptable fit, χ^2^(63) = 169.63, CFI = 0.965, TLI = 0.960, RMSEA = 0.077, and SRMR = 0.048. Changes in model fit indices relative to the configural model were negligible (ΔCFI = 0.001, ΔRMSEA = 0.004), supporting metric invariance.

Scalar invariance was then examined by additionally constraining item intercepts to be equal across groups. The scalar invariance model demonstrated acceptable fit, χ^2^(70) = 191.66, CFI = 0.960, TLI = 0.959, RMSEA = 0.078, and SRMR = 0.055. Changes in model fit indices between the metric and scalar models remained within the recommended thresholds (ΔCFI = 0.005, ΔRMSEA = 0.001), supporting scalar invariance.

### Reliability

Internal consistency reliability was assessed using Cronbach’s alpha and McDonald’s omega in both sample 1 and sample 2. The results indicated excellent internal consistency, with Cronbach’s alpha ranged from 0.93 to 0.94, and McDonald’s omega ranged from 0.93 to 0.94. Test–retest reliability was evaluated using the intraclass correlation coefficient (ICC). The ICC was 0.94 (95% CI [0.93, 0.95], *n* = 349), indicating excellent temporal stability over the 2-week interval.

## Discussion

The purpose of this research was to produce a Chinese version of the Perceived Variety Support in Physical Education Scale (PVSPES) and to evaluate its measurement qualities within a sample of Chinese adolescent students. The results confirmed that the scale is reliable. These results support the use of this scale to assess perceived variety support in Chinese PE settings. In addition, this study extends the concept of perceived variety support to a new cultural and educational context. The concept retains its core meaning in Chinese PE.

Exploratory factor analysis and confirmatory factor analysis supported a unidimensional structure of the scale. Although the PVSPES items refer to different sources of variety in PE, the results suggest that students may perceive these elements as part of one broader variety-supportive learning environment. In other words, Chinese high school students may not distinguish sharply between different sources of variety, but instead form an overall judgment about whether their PE lessons provide varied experiences. These findings indicate that students perceive variety support in physical education as a general feature of the instructional environment. This factor structure is consistent with the original conceptual framework of the PVSPES proposed by Eather et al. (2022), suggesting similar understandings of variety support among Chinese adolescents. The strong factors also indicate that each item makes a substantial contribution to the construct. Overall, these findings further confirm that the scale has strong internal consistency and a robust latent structure. Future studies may further examine whether these aspects can form distinct subdimensions in larger or more diverse samples.

Furthermore, the scale showed excellent internal consistency. High values of Cronbach’s alpha supported this result. High values of McDonald’s omega also confirmed this finding. The scale also demonstrated strong test–retest reliability. A high intraclass correlation coefficient indicated stable scores over time. These reliability indicators meet strict standards for psychological and educational measurement. The results show that the Chinese version of the PVSPES produces consistent outcomes. These results suggest that the scale may be suitable for preliminary research use in similar samples.

An important contribution of this study is the establishment of measurement invariance across gender. Multi-group confirmatory factor analyses supported configural invariance. The analyses focused on male and female adolescents also supported metric and scalar invariance.

More specifically, configural invariance indicates that boys and girls understood the general factor structure of the scale in a similar way. Metric invariance suggests that the items contributed to the latent construct comparably across gender groups, while scalar invariance indicates that observed scores can be compared more meaningfully between boys and girls. The results also show that factor loadings and item intercepts are equivalent across gender. These findings indicate that the scale functions in a similar way for male and female students (Putnick and Bornstein, 2016). The scale therefore allows valid comparisons of perceived variety support between genders.

To the best of our understanding, this research represents the earliest effort to culturally adapt and empirically validate the PVSPES for use within a Chinese setting. Beyond extending the scale to a new linguistic and cultural setting, the present findings provide psychometric evidence for assessing perceived variety support as a psychologically meaningful construct in physical education. From a self-determination theory perspective, perceived variety support may serve as an important contextual factor that supports motivational processes in PE. Practically, the scale may help PE teachers identify students’ perceptions of variety in teaching methods, equipment, activity goals, and lesson formats, thereby informing psychologically supportive instructional practices. Previous research has suggested that a supportive physical education environment with greater perceived variety in teaching content, methods, and learning opportunities may enhance students’ engagement and thereby promote their socialization and interpersonal development within school contexts (Buyrukoğlu et al., 2023).

The Chinese version of the PVSPES can also serve as a valid instrument for testing theoretical frameworks in physical education. Researchers can use this scale to examine self-determination theory in the Chinese context. The scale provides a clear way to measure individuals’ perceived level of variety support, enabling researchers to explore the relationships between diversity support and key educational outcomes.

## Limitations and perspectives for future research

Although the findings contribute meaningfully to the literature, the research still faces certain constraints. One notable limitation is the exclusive use of participant self-report measures. This method may introduce social desirability bias and recall bias, which can affect data accuracy. In addition, because all data were collected through self-report questionnaires, common method bias may have been present. Second, participants were recruited through convenience sampling from six high schools in Shanghai. The generalizability of the findings to all Chinese adolescents should be interpreted with caution. Future studies should further validate the Chinese PVSPES using larger and more diverse samples from different regions, school types, and age groups. Third, the sample consisted only of high school students. This sample choice limits conclusions for other age groups. Future studies should test the scale with younger students. Researchers should include elementary and junior high school populations. Fourth, participants were recruited through convenience sampling, a procedure that may weaken the extent to which the sample reflects the target population. Future studies are encouraged to employ probability sampling techniques to improve external validity and strengthen the robustness of conclusions. Lastly, the validity evidence provided in this study remains incomplete. The present study mainly examined structural validity, internal consistency, test–retest reliability, and gender invariance, while criterion-related validity and broader convergent and discriminant validity evidence were not fully examined. The statistical results should also be interpreted with caution, as multivariate normality was not formally tested and the RMSEA value was close to the upper boundary of acceptable fit. Future studies should provide more comprehensive validity evidence and use additional statistical approaches to further examine the robustness of the Chinese PVSPES.

## Conclusion

This study translated the PVSPES into Chinese and provided initial evidence for its validity and reliability among Chinese high school students. Further studies using more diverse and representative samples are needed to examine the broader applicability of the Chinese PVSPES.

## Data Availability

The raw data supporting the conclusions of this article will be made available by the authors, without undue reservation.
